# Succession of Fungal Community during Outdoor Deterioration of Round Bamboo

**DOI:** 10.3390/jof9060691

**Published:** 2023-06-20

**Authors:** Xiaojiao An, Shuaibo Han, Xin Ren, John Sichone, Zhiwei Fan, Xinxing Wu, Yan Zhang, Hui Wang, Wei Cai, Fangli Sun

**Affiliations:** 1 School of Chemical and Materials Engineering, National Engineering & Technology Research Center for the Comprehensive Utilization of Wood-Based Resources, Zhejiang A&F University, Hangzhou 311300, China; axj624@stu.zafu.edu.cn (X.A.); rx@stu.zafu.edu.cn (X.R.); johnsichone450@gmail.com (J.S.); fzw@stu.zafu.edu.cn (Z.F.); xinxingwu@zafu.edu.cn (X.W.); zhangy@iccas.ac.cn (Y.Z.); wanghui@zafu.edu.cn (H.W.); 2 Microbes and Insects Control Institute of Bio-based Materials, Zhejiang A&F University, Hangzhou 311300, China; 3 Anji Zhujing Bamboo Technology Co., Ltd., Huzhou 313300, China; davidcai7010052023@163.com

**Keywords:** bamboo deterioration, biodiversity, fungal community

## Abstract

Bamboo’s mechanical and aesthetic properties are significantly influenced by fungi. However, few studies have been conducted to investigate the structure and dynamics of fungal communities in bamboo during its natural deterioration. In this study, fungal community succession and characteristic variations of round bamboo in roofed and unroofed environments over a period of 13 weeks of deterioration were deciphered using high-throughput sequencing and multiple characterization methods. A total of 459 fungal Operational Taxonomic Units (OTUs) from eight phyla were identified. The fungal community’s richness of roofed bamboo samples showed an increasing trend, whereas that of unroofed bamboo samples presented a declining trend during deterioration. Ascomycota and Basidiomycota were the dominant phyla throughout the deterioration process in two different environments: Basidiomycota was found to be an early colonizer of unroofed bamboo samples. Principal Coordinates Analysis (PCoA) analysis suggested that the deterioration time had a greater impact on fungal community variation compared to the exposure conditions. Redundancy analysis (RDA) further revealed that temperature was a major environmental factor that contributed to the variation in fungal communities. Additionally, the bamboo epidermis presented a descending total amount of cell wall components in both roofed and unroofed conditions. The correlation analysis between the fungal community and relative abundance of three major cell wall components elucidated that Cladosporium was negatively correlated with hemicellulose in roofed samples, whereas they presented a positive correlation with hemicellulose and a negative correlation with lignin in unroofed samples. Furthermore, the contact angle decreased during the deterioration process in the roofed as well as unroofed samples, which could arise from the degradation of lignin. Our findings provide novel insights into the fungal community succession on round bamboo during its natural deterioration and give useful information for round bamboo protection.

## 1. Introduction

Climate change is the most significant global health threat in the twenty-first century, which results in serious environmental problems, such as extreme weather, desert expansion, glacial retreat, and ecological collapse [1]. Substituting renewable and low-carbon-intensity materials for fossil fuel-intensive materials can make a significant contribution to the mitigation of climate change [2]. Bamboo, a vital and renewable non-wood forest resource, is abundant in many tropical and subtropical regions, particularly in China, which holds 29.2% of the global bamboo area [3]. Bamboo offers numerous advantages, including rapid growth, simple cultivation, high yield, outstanding mechanical performance, and sustainable utilization, which has led to its widespread use in household products, furniture manufacturing, and housing construction [4]. The bamboo industry is deeply intertwined with people’s daily lives and plays a significant role in the economy of many Asian countries, such as India and China [5]. As a green and sunrise industry, the bamboo sector has experienced remarkable growth, making it an essential contributor to climate change mitigation and sustainable development [6,7,8].

Round bamboo is a raw form of bamboo, consisting of a hollow cylindrical culm divided into sections by nodes, and on its exterior wall are bamboo epidermis, cortex, ground tissue, and a vascular bundle. In commercial applications, the culm wall is divided into bamboo green, bamboo yellow, and a bamboo membrane [9]. Unlike engineered bamboo boards, which are manufactured from bamboo units such as bundles, strips, and slivers, round bamboo culms retain the natural texture, shape, and mechanical structure of raw bamboo materials [10]. Round bamboo is also the most historically used form of bamboo material due to its attractive appearance, high strength, and bending modulus, and has gained growing attention in building, decorating, and furnishing [9,11]. The round bamboo construction is more than a place for people to rest; it is also an integral part of the distinctive landscape line of a cultural tourist scenic location, which is endowed with both aesthetic and cultural significance.

However, mildew and decay are the primary threats to round bamboo structures and their visual characteristics, drastically reducing their service life [12]. Mildew is caused by mold fungi, and their susceptibility to mold development is often affected by both the specific bamboo material utilized and environmental factors [13]. Fresh round bamboo is a rich source of organic nutrients, including 1.5–6% protein, 2% soluble sugar, 2.2–5.18% starch, 2.18–3.55% fat and wax, and over 30% water [14]. These nutrients, especially sugar, and starch, act as ideal food for the growth of mold fungi. Stains caused by mold fungi have a significant impact on the aesthetic characteristics of round bamboo, which severely restrict its application, leading to huge economic losses and the waste of bamboo resources. In addition, mold also has significant adverse effects on human health, especially in indoor environments. When mold spores are present in sufficient amounts, certain types of mold fungi could potentially cause severe allergic reactions [15]. *Stachybotrys chartarum*, also known as toxic black mold, is often found in cellulose-rich building materials, which is able to produce highly toxic macrocyclic trichothecene mycotoxins that are capable of damaging DNA, posing a significant public health risk [16]. Damage by decay fungi is another severe problem during the storage and utilization of round bamboo. These fungi are functionally classified as white, brown, and soft-rot types. Generally, white and brown rot are largely Basidiomycete fungi that play crucial roles in the decay of both cellulose and lignocelluloses, whereas soft-rot fungi mainly target cellulose [17]. *Schizophyllum commune*, a fungus that occurs on every continent other than Antarctica, causes the white-rot decay of bamboo, acting as a ubiquitous saprophyte as well as an opportunistic pathogen [18]. Kim also reported that *Trametes versicolor* and *Arthrinium arundinis* were the two most degrading fungi among the six selected fungi, causing weight losses as high as 21.6% and 17.9%, respectively [19].

Fungal succession, which refers to different species that become dominant at different points in time, is one of the most widespread and universal patterns of community assembly and activity in the ecosystem’s ecology [20]. Although fungal succession on various plant substrates has been well explored [21,22,23,24,25], it has received little attention when it comes to bamboo. Zhou and Hyde [26] placed several bamboo culms in terrestrial habitats in Hong Kong and investigated their fungal succession on bamboo via culturing on a medium and observing fruit bodies under a microscope in the laboratory. They suggested that the fungal community on bamboo could be categorized into early, middle, and later-stage colonizers, and seasonality has an effect on fungal succession, as more fungi were present during the wet season. However, since any medium or cultural condition is more or less “selective”, it makes it difficult to observe all microorganisms using culture-dependent methods [27]. High-throughput sequencing (HTS) techniques are considered indispensable culture-independent approaches that can overcome the limitations of the classical culture-based approach and have been extensively used to study fungal community succession [28,29,30,31,32,33].

In the present study, we investigated the dynamic changes of fungal communities on round bamboo via high-throughput sequencing combined with multivariate data analysis. Additionally, the variation in chemical components, morphology, and other properties of the bamboo epidermis in different deterioration stages was analyzed via high-performance liquid chromatography (HPLC), scanning electron microscopy (SEM), X-ray Photoelectron Spectroscopy (XPS), and contact angle measurements. The main objective of the present study was to address the following questions: (1) How did fungi communities dynamically change during outdoor bamboo deterioration? (2) How did the characteristics of round bamboo change during outdoor deterioration, and what is the relationship between fungal succession and bamboo characteristics? (3) What were the main factors that affected the fungal community’s structure and diversity during the deterioration of round bamboo exposed outdoors?

## 2. Materials and Methods

### 2.1. Study Site and Bamboo Sampling

This study was conducted at the campus of Zhejiang A&F University, Lin’an District, Hangzhou (119°44′ E, 30°15′ N). The area was located in the northwestern Zhejiang province and the south-central part of the Yangtze River Delta. As a typical area of southern China, this district also experiences extremely warm and wet periods in the middle of each year. This period is called the Meiyu season, which generally starts in early June and ends in mid-July [34]. The Meiyu season is one of three periods of heavy rainfall in China, and the precipitation during this period is mostly continuous and stable [35]. The favorable temperature and relatively high humidity during this season encourage the formation of mold and rot not only on fabrics but on wood materials as well. 

**Figure 1:**
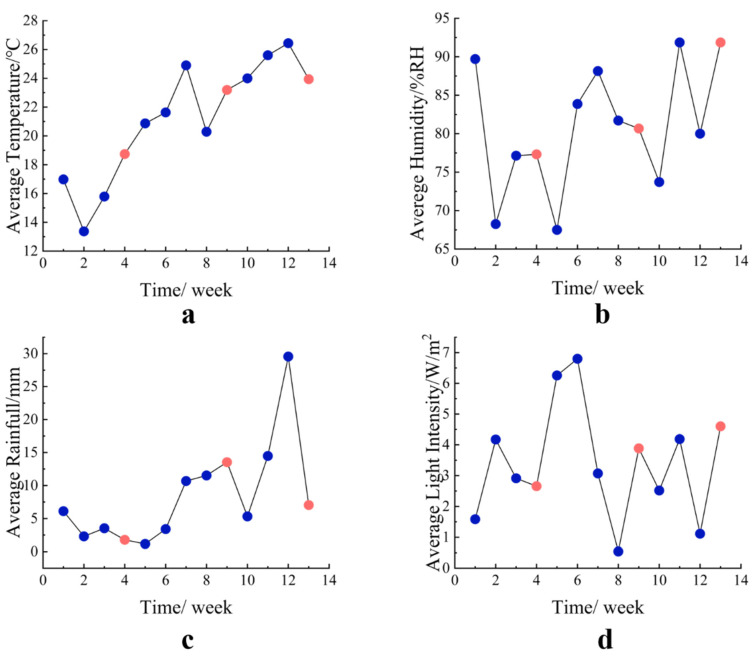
Changes in temperature (**a**), humidity (**b**), rainfall (**c**), and light intensity (**d**) during the round bamboo deterioration process. Pink dots represent the environmental factors of sample collection time, and blue dots represent the weekly environmental factors during the deterioration process.

Four-year-old bamboo (*Phyllostachys iridescens*) was collected from the bamboo forest in Anji County, Zhejiang, China (119°14′ E, 30°53′ N) in 2021. Bamboo culms around 10 cm in diameter were selected from the bottom up at 2–4 m. Subsequently, the culms were cut into 30 cm long round bamboo segments and sterilized with an 80% alcoholic solution. According to the Chinese National Standard GB/T 27651-2011 [36], two biologically hazardous conditions for round bamboo segments C3.1 (exterior, above-ground, roofed) and C3.2 (exterior, above-ground, unroofed) were designed. Bamboo segments were placed on the rack, as shown in Figure S1, with the bottom of the rack appearing 20 cm above the ground. We set roofed (Q) and unroofed (QY) groups to simulate C3.1 and C3.2 conditions. The main difference between the Q groups and QY groups was that Q groups were roofed with plastic sheeting to avoid exposure to rain, whereas QY groups were exposed to rainfall directly. Bamboo samples were collected at week_4, week_9, and week_13 of the deterioration process, which represented the initial, mid-term, and later stages of the Meiyu season. Three biological replicates were sampled for each stage. For each sample, the middle zone of the bamboo segments with a depth of 1 mm was selected for DNA extraction to avoid fungal contamination from both ends. The data on temperature, humidity, illumination, and rainfall during this process were provided by the Meteorological Bureau of Lin’an District (Figure 1), where the experimental site was located.

### 2.2. Fungal Community Analysis

#### 2.2.1. DNA Extraction and Sequencing

The DNA of the fungi present on the bamboo samples was extracted using a TGuide S96 Magnetic DNA Kit (Tiangen Biotech (Beijing) Co., Ltd., Beijing, China) based on the manufacturer’s instructions. The concentration and purity of extracted DNA were measured using the Qubit dsDNA HS Assay Kit and Qubit 4.0 Fluorometer (Invitrogen, Thermo Fisher Scientific, Hillsboro, OR USA). ITS1 (5′-CTTGGTCATTTAGAGGAAGTAA-3′) and ITS4 (5′-TCCTCCGCTTATTGATATGC-3′) were used as fungal full-length primers [37]. Sample-specific barcode sequences were tailed on both forward and reverse primers to enable multiplexed sequencing.

PCR was performed using the buffer system of a KOD One PCR Master Mix (TOYOBO Life Science, San Jose, CA, USA) under the following conditions: initial denaturation at 95 °C for 2 min; 25 cycles at 98 °C for 10 s, 55 °C for 30 s and 72 °C 90 s; and final extension at 72 °C for 10 min. The PCR products were purified with Agencourt AMPure XP Beads (Beckman Coulter, Indianapolis, IN, USA) and quantified using the Qubit dsDNA HS Assay Kit and Qubit 4.0 Fluorometer (Invitrogen, Thermo Fisher Scientific, Hillsboro, OR, USA) [38]. The amplicons were pooled in equal amounts after individual quantification. A SMRTbell Express Template Prep Kit 2.0 was used to prepare SMRTbell libraries from amplified DNA. The sequencing of amplicon libraries was performed on a single PacBio Sequel II 8M cell using the Sequel II Sequencing Kit 2.0 [39].

#### 2.2.2. Sequence Data Processing

In this study, BMK Cloud (Biomarker Technologies Co., Ltd., Beijing, China) was used for bioinformatics analysis. Raw sequencing reads were processed using the SMRT Link software (version 8.0) to achieve circular consensus sequencing (CCS) reads. The CCS sequences were assigned to the corresponding samples based on their barcodes with the aid of the lima software (version 1.7.0). The Cutadapt quality control process (version 2.7) was used to exclude CCS reads that contained no primers and were read exceeding the length range (1200–1650 bp). Clean reads were obtained by detecting and removing chimera sequences using the UCHIME algorithm [40]. USEARCH [41] (version 10.0) was used to cluster sequences with a similarity ≥97% into the same operational taxonomic unit (OTU), and OTUs were filtered with redundance <0.005%. The taxonomy annotation of OTUs was performed based on the UNITE (version 7.1) database [42]. Sequencing depth was investigated by plotting rarefaction curves.

#### 2.2.3. Statistical Analysis

The alpha diversity, including ACE, Chao1, Shannon, and Simpson indices, were calculated and displayed by QIIME2 and R software (version 4.2.0) [43], respectively. For analyses of beta diversity, an unweighted pair-group method with arithmetic means (UPGMA), heat maps, and principal coordinate analysis (PCoA) were performed using QIIME. To explore the dissimilarities of the microbiome among different factors, a redundancy analysis (RDA) was performed in R using the package ‘vegan’. The FUNGuild database was used to assign fungal communities to functional guilds. (e.g., parasitic, phytopathogenic, and saprophytic).

### 2.3. Characteristics of Bamboo

#### 2.3.1. Chemical Composition Determination

The surface of bamboo culms with a depth of 1 mm was scraped and ground into 80 mesh powder. The bamboo powder was dried at a rising temperature: 60 °C for 2 h, 80 °C for 2 h, and 105 °C until constant weight. A Soxhlet extractor was used to analyze the extractive content of the bamboo sample using an ethanol-benzene solution (ethanol: Benzene = 1:2, *v*/*v*) for 8 h. The chemical composition of the bamboo samples, including cellulose, xylan, arabinan, acid-soluble lignin (ASL), acid-insoluble lignin (AIL), and ash, were determined using the procedure proposed by the National Renewable Energy Laboratory (NREL). In total, 0.3 g of each sample was soaked in 72% (*w*/*w*) H_2_SO_4_ at 30 °C for 1 h, followed by 4% (*w*/*w*) H_2_SO_4_ hydrolysis at 121 °C for another hour. Then, the acid hydrolysis solution was analyzed by HPLC to calculate the carbohydrate contents. Specifically, the sums of xylan and arabinan were employed to represent the hemicellulose contents within the bamboo samples. To determine the ash content, 2 g of each sample was weighed and completely burned at 575 °C for 12 h. The ash content was calculated by dividing the burned residue’s dry weight by the original bamboo powder sample [44,45].

#### 2.3.2. XPS Analysis

The scraped bamboo epidermis, after being finely grounded into 100 mesh, was determined on a Thermo Scientific (Hillsboro, OR, USA) K-Alpha XPS system with a monochromatic Al KαX-ray source (hv = 1486.6 eV). A binding energy (BE) range of 0 to 1350 eV and a pass energy of 150 eV were used to collect the survey spectra. High-resolution scans of the examined peaks were collected at pass energy of 50 eV. All spectra were obtained using a 400 nm diameter analysis area. The peaks of all the samples were calibrated with the BE for C1s at 284.6 eV. The C and O peaks were separated into subcomponents using the Lorentzian–Gaussian distribution after background subtraction according to Shirley using an XPS peak 4.1 software package.

#### 2.3.3. SEM Observation

The changes occurring on the bamboo epidermis were examined using a scanning electron microscope (SEM; TM3030, HITACHI, Tokyo, Japan). Bamboo culms were cut into small pieces. Then, the epidermis was collected and fixed on the aluminum sample table with carbon tape. SEM scanning was carried out at 15 kV voltages after 10 nm of gold film coating under a vacuum.

#### 2.3.4. Contact Angle Measurements

The hydrophobicity of the bamboo surface was measured by contact angle analysis using an OCA50AF contact angle measuring instrument (Dataphysics, Stuttgart, Germany). The volume of the deionized water droplet was fixed at 5 μL, and the test time was 5 s.

## 3. Results and Discussion

### 3.1. Richness and Diversity Assessment

High-throughput sequencing was used to assess the fungal communities present on the bamboo surface at different stages. In total, 459 Operational Taxonomic Units (OTUs) were obtained from six samples at three different stages. The OTUs belonged to 8 phyla, 26 classes, 61 orders, 117 families, and 178 genera. The Shannon curves of multi-samples tended to be flat, indicating that the sequencing results adopted were sufficient to reflect the whole fungal diversity (Figure S2).

#### 3.1.1. Alpha Diversity Analysis

Alpha diversity is one of the most important characteristics of a microbial community, indicating the richness of species and patterns of community distribution. Alpha diversity can be reflected by many indexes, including Chao1, ACE, Shannon, and Simpson. Chao1 and ACE indexes present community richness, while Shannon and Simpson indicate community evenness.

**Figure 2:**
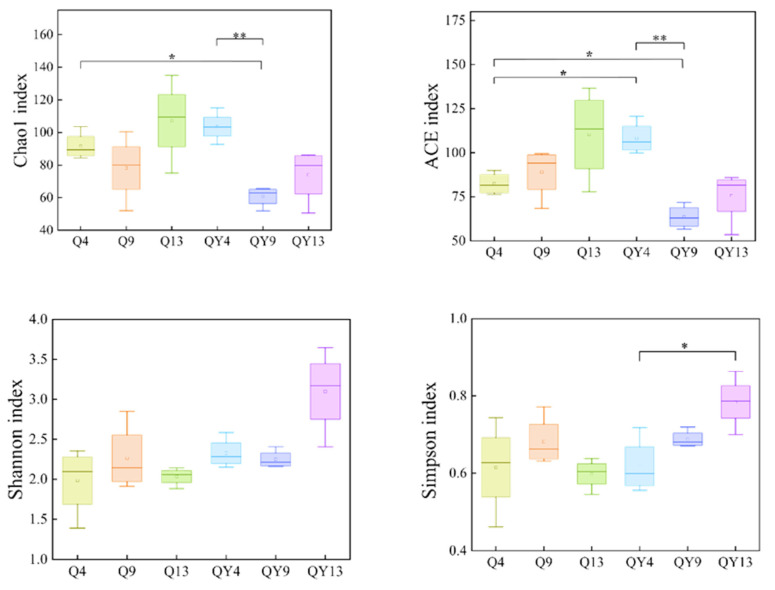
The Chao1 index, ACE index, Shannon index, and Simpson index of the fungal community during different stages of the round bamboo deterioration process. The asterisks indicate statistically significant differences between stages at the 0.05 probability level, as determined by the Duncan test (* *p* < 0.05, ** *p* < 0.01).

In this study, the Chao1 and ACE indexes of roofed fresh bamboo after exposure for four (Q4), nine (Q9), and thirteen (Q13) weeks showed an increasing trend during outdoor deterioration, whereas the unroofed group (QY4, QY9, and QY13) experienced a decreasing trend during the same period (Figure 2). Moreover, compared to the QY4 group, the Chao1 and ACE indexes of the QY9 group showed a highly significant decrease (*p* < 0.01). Bouskill proposed that increased rainfall may enhance anoxic conditions and nutrient leaching and, thus, inhibit microbial activities in humid ecosystems [46]. The bamboo samples of the QY groups underwent continuous rainfall and water leaching during the experimental period. Water leaching removed the starch, carbohydrates, and other water-soluble substances in the bamboo, and thus the decay and mold resistance of bamboo was improved [47]. Therefore, nutrient leaching may be the main reason for the decreasing Chao1 and ACE indexes of QY groups. In addition, the Shannon and Simpson indexes of the roofed groups (Q4, Q9, and Q13) did not show a clear change. Similar results were reported by Niu et al. (2021), who reported that rainfall reduced community richness but had little impact on the community diversity of fungi [48]. Interestingly, the QY13 group had the highest Shannon and Simpson indexes among all the groups and had a significant increase compared with the QY4 group (*p* < 0.05). Considering the relatively low richness, we speculated that the community evenness of the QY13 group was higher than that of the other groups. In addition, fungal spores were ubiquitous in the air, especially in humid environments, and enhanced fungal spores could be released into the air due to the mechanical disturbance caused by raindrops [49]. Moreover, raindrops are rich in fungal species [50] as well, and biological particles, including fungal spores, are abundant in cloud ice crystals and precipitation residues [51]. These reasons could also contribute to the diversity index increase in the QY13 group.

**Figure 3:**
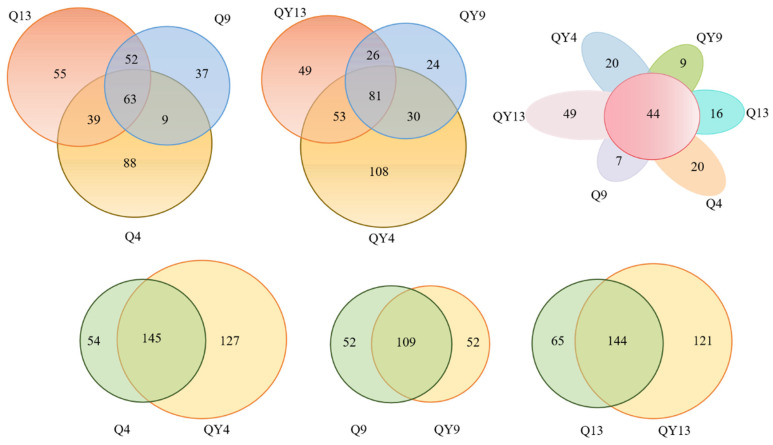
OTU-level Venn diagrams within the same group and OTU-level Venn diagrams for different groups at the same time period during the round bamboo deterioration process.

Venn diagrams can intuitively indicate the similarity and overlap among different groups. As can be seen in Figure 3, the Q4, Q9, and Q13 groups contained 63 shared OTUs, as well as 88, 37, and 55 unique OTUs, respectively. Similarly, the QY4, QY9, and QY13 groups contained 81 shared OTUs, as well as 108, 24, and 49 unique OTUs, respectively. The number of overlapping OTUs among all the groups (Q4, Q9, Q13, QY4, QY9, and QY13) was 44. In addition, the overlapping OTUs between Q4 and QY4, Q9 and QY9, Q13 and QY13 were 145, 109, and 144, respectively.

#### 3.1.2. Beta Diversity Analysis

**Figure 4:**
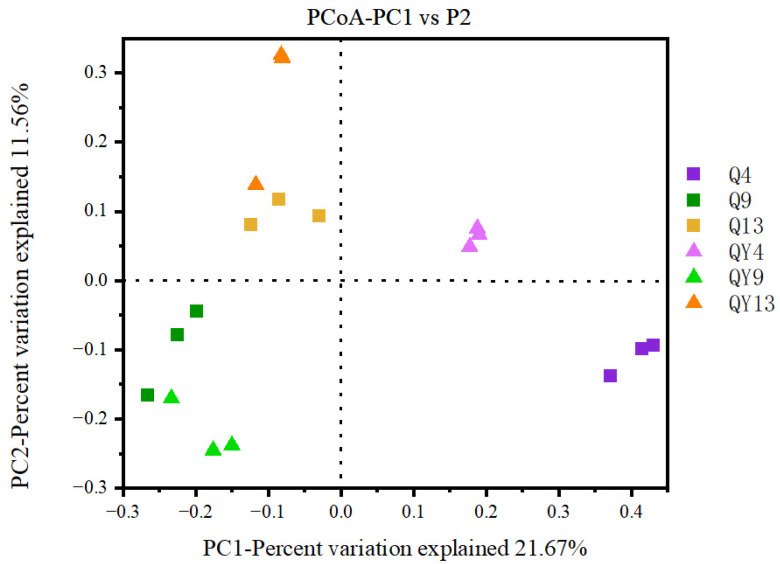
Principal coordinate analysis (PCoA) of the fungal communities during round the bamboo deterioration process. PCoA distances were based on the Jaccard distance algorithm at the OTU level.

Principal coordinate analysis (PCoA) was performed to identify the fungal community profiles at each deterioration time. As shown in Figure 4, the differential contribution rate of the microbial community structure for the first two principal components (PC1 and PC2) was 21.67% and 11.56%, respectively. The distribution of samples on the PCoA plot was obviously different. Q4 and QY4 groups were located on the positive side of PC1, whereas Q9 and QY9 groups were distributed on the negative side of PC2. Notably, the distribution of the three roofed groups (Q4, Q9, Q13) was discrete and did not gather together, while the samples of the groups collected at the same time (e.g., Q4 and QY4, Q9 and QY9, Q13 and QY13) tended to be clustered together. UPGMA dendrograms of the fungal community showed similar results to the PCoA plot. Samples with the same collection time were frequently clustered together (Figure S3). These results indicate that different deterioration times make caused a greater contribution to the variation in the fungal community in comparison with roofed or unroofed environments, and accordingly, time span had a greater impact on the succession of the fungal community of round bamboo. In addition, the three repetitive samples of group Q4 were clustered into one branch, while three replicates of QY4 were clustered into another branch in UPGMA dendrograms, which illustrated that the difference between these two groups was mainly due to rainfall. Meanwhile, Q4 and QY4 groups gathered together and formed a distinct branch from the other groups, which indicated that the fungal community varied drastically during the early stage of the bamboo deterioration process. The meteorological data analysis showed that except for average humidity, the gap in the average temperature, rainfall, and illumination between week_4 and week_9 was much greater than that between week_9 and week_13.

### 3.2. Microbial Community Composition

**Figure 5:**
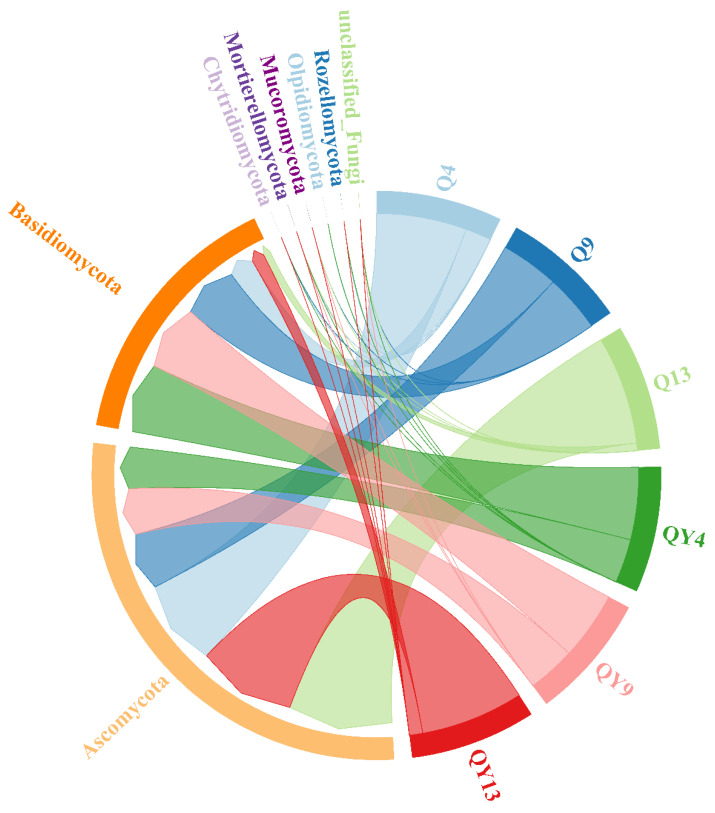
Relative abundance of fungal communities at the phylum level during the round bamboo deterioration process.

The distribution of fungi at the phylum level on the surface of outdoor bamboo is shown in Figure 5. The most significant feature was the massive colonization of bamboo by Basidiomycota and Ascomycota. Among the roofed groups, the relative abundance of Basidiomycota was 22.33%, 49.71%, and 6.51% in the Q4, Q9, and Q13 groups, respectively. In the unroofed groups, the relative abundance of Basidiomycota was 62.19%, 58.69%, and 11.47% in the QY4, QY9, and QY13 groups, respectively. Ascomycota was another dominant phylum in the fungal communities comprising 77.43%, 50.22%, and 93.35% in the Q4, Q9, and Q13 groups, respectively. Whereas the relative abundance of Ascomycota in the unroofed groups was 37.63%, 41.46%, and 88.04%, respectively. When comparing samples collected at the same time (Q4 vs. QY4, Q9 vs. QY9, Q13 vs. QY13), the relative abundance of Basidiomycota in the roofed groups was lower compared to that of the unroofed groups. On the contrary, the relative abundance of Ascomycota in the roofed groups was higher than that of the unroofed groups during the corresponding period of sample collection. Considering that the main difference between the roofed and unroofed samples was continuous precipitation, we speculated that rainfall was able to increase the relative abundance of Basidiomycota but decrease that of Ascomycota. This result was consistent with the previous study by Zhao et al. (2018), who found that water addition increased the relative abundance of Basidiomycota and decreased that of Ascomycota in the soil microbial community composition [52]. Niu et al. (2021) also found that the proportion of Ascomycota was higher on non-rainy days than on rainy days, whereas the percentage of Basidiomycota was much lower on non-rainy days than on rainy days [49]. Ascomycetes and Basidiomycetes were the most abundant groups in the soil fungal community [53]. Generally, Ascomycota mainly contributes to the early stages of lignocellulose degradation, and Basidiomycota mainly contributes to the late stages of lignocellulose degradation [54]. However, we found that the relative abundance of Basidiomycota in the unroofed groups reached its highest level at week_4 and showed a declining trend during the deterioration process. It has been reported that wood-decay basidiomycetes prefer an acidic condition, whereas ascomycetes prefer slightly alkaline environments [55]. The XPS analysis of unroofed groups showed that C4, characterizing the organic acid, increased in week 4 and then declined gradually to the same value as the control. As for the roofed groups, C4 increased sharply at week 9 and then declined rapidly to a low value, which corresponded to the variation in the relative abundance of Basidiomycota (Figure S4). Therefore, we speculated that the acid micro-environment on the unroofed round bamboo surface could lead to a high relative abundance of Basidiomycota at the initial stage of deterioration. Other phyla, such as Chytridiomycota, Mortierellomycota, Olpidiomycota, and Rozel-lomycota, were also recognized on roofed or unroofed bamboo samples in low abundance. Chytridiomycota, also known as chytrids, is one of the earliest diverging fungal lineages [56] and is widely distributed in soil and aquatic environments [57]. Recent research has revealed that Chytridiomycota were also found in many extreme environments, such as the desert [58], polar regions [59], and hydrothermal vents [60]. Chytridiomycota is able to penetrate some resistant structures and digest pollen, cellulose, chitin, and keratin to dissolved organic matter and dissolved inorganic matter [61], which play pivotal roles in the decomposition of particulate organic matter (POM) [62]. The phylum Mortierellomycota was proposed by Leho [63] based on phylogenies and divergence time. It has been reported that Mortierellomycota participates in the early stages of organic residue decomposition, releasing nutrients into the soil through the breakdown of plant residues [64] Olpidiomycota was introduced by Doweld [65] to accommodate Olpidiales, which was accepted in Chytridiomycetes [66]. There was only one class, one order, one family, and four genera in Olpidiomycota, and members of Olpidiomycota exhibited a wide range of life modes as saprobes and parasites [67]. Rozellomycota was located near the phylogenetic root of the Kingdom Fungi and existed ubiquitously from terrestrial to aquatic ecosystems [68]. All known members of Rozellomycota are obligate pathogens of various other eukaryotes, such as amoebae, algae, and other fungi [69]. It is well known that some minor taxa may play a critical role in the microbial community’s succession. However, the function of those minor taxa in round bamboo deterioration is still unknown and needs further investigation.

**Figure 6:**
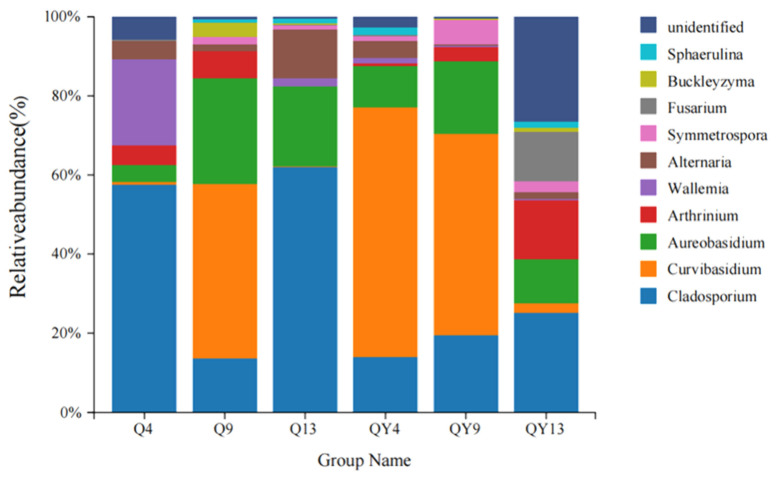
Relative abundance of fungal communities in bamboo outdoor deterioration at the genus level.

At the genus level, the dominant taxa found included representatives of the genera *Cladosporium*, *Curvibasidium*, *Aureobasidium*, *Wallemia*, *Alternaria*, *Symmetrospora*, *Fusarium*, *Buckleyzyma*, and *Sphaerulina* (Figure 6). *Cladosporium* dominated the deterioration process in both the roofed and unroofed groups, especially in Q4 (55.80%) and Q13 (58.76%). *Cladosporium* is one of the largest genera of the dematiaceous hyphomycetes fungus, and their adaptation to different environments allows them to have a broad lifestyle [64]. It is noted that *Cladosporium* spp. is also widely distributed in bamboo materials, e.g., decayed leaves [70], dead culms [71], and moso bamboo seeds [72]. *Curvibasidium* was the predominant fungal genus in Q9 (42.42%), QY4 (58.49%), and QY9 (48.56%), respectively. This genus was a yeast-like fungus and was proposed by Sampaio et al. (2004) to accommodate the sexual states of two yeasts of the Microbotryomycetes, *C. cygneicollum* Sampaio and Golubev and *C. pallidicorallinum* Sampaio and Golubev. The species of this genus *Curvibasidium* were widely distributed in various environments, such as marine sediments and white grapevine [73,74,75,76]. However, to our best knowledge, no research has reported on the colonization of *Curvibasidium* on bamboo. It has been reported that *Curvibasidium pallidicorallinum* is able to produce polygalacturonase, making it a candidate for enhancing the breakdown of berries’ cell walls during fermentation [77]. It is noteworthy that *Wallemia* showed a high relative abundance in Q4 (21.01%), while it almost disappeared in other groups. The genus *Wallemia* belongs to the order Wallemiales (Wallemiomycotina, Basidiomycota) and comprises the most xerotolerant, xerophilic, and also halophilic species worldwide [78]. *Wallemia sebi* is the most-studied species among the *Wallemia*, and it can grow in a relatively low water-activity (below 0.85) environment [79]. Similarly, *W. muriae* and *W. ichthyophaga*, another two species of *Wallemia*, have the obligatory demand for lowered active water (a_w_) in their habitats. Therefore, *Wallemia* spp. were found in various osmotically challenging environments, such as dry foods, salt crystals, and indoor and outdoor air [77]. Because of the continuous rainfall and increasing humidity, bamboo samples of QY4, QY9, QY13, Q9, and Q13 groups were supposed to have a higher water activity than that of Q4, making them unsuitable habitats for *Wallemia*.

### 3.3. The Correlation between Microbial Community Structures and Environmental Factors

**Figure 7:**
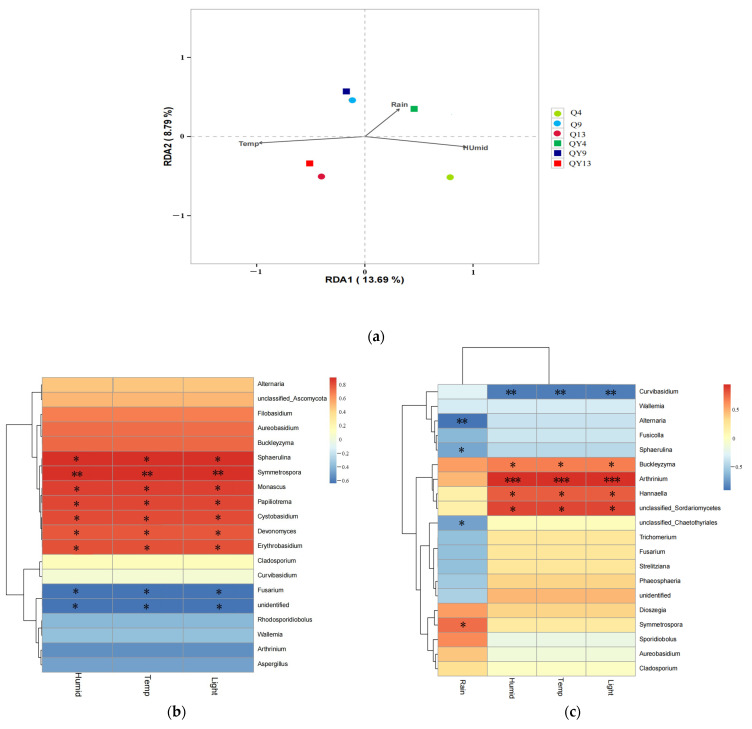
Relationship between fungal communities and environmental factors revealed by redundancy analysis (RDA) (**a**). Heat map of the correlation between fungal communities and environmental factors at the genus level of the roofed (**b**) and unroofed groups (**c**). The colors in the heat maps indicate the Spearman correlation coefficient r which ranges from −1 to 1; r < 0, negative correlation (blue); r > 0, positive correlation (red); Asterisks represent significance level: * *p* < 0.05, ** *p* < 0.01, and *** *p* < 0.001.

RDA was performed to illustrate the relationship between environmental factors and fungi communities (Figure 7a). The results indicated that among the four environmental factors collected, three parameters were significant in explaining the fungal community’s structure variability (*p* < 0.01). However, they only explained 22.48% of this variability in total. Axis 1 explained 13.69% of this variation, and Axis 2 explained another 8.79%. The major factor that explained most of the variation in the bamboo fungal community composition was temperature, followed by humidity and rainfall. Temperature plays a critical role in the colonization and growth of microorganisms, which could explain why time span, rather than roofed or unroofed conditions had a greater impact on the succession of the fungal community on round bamboo, as concluded from the principal coordinate analysis. The chemical reactions occurring in vivo during microbial growth were mostly accomplished by the catalysis of specific enzymes. Each enzyme had an optimal enzymatic reaction temperature, and a higher or lower temperature was able to limit the enzymatic reaction rate and, ultimately, the synthesis of cellular materials. The fluidity of the cell membrane was also affected by temperature. A relatively high temperature endowed the high fluidity of the cell membrane, thus improving the absorption of nutrients and the secretion of metabolites. Generally, the optimal temperature for fungal growth was 20–30 °C, which was within the temperature range after week_4 in this study. In addition, many interactions between the fungi and other decomposers of the microbial community were influenced by temperature [80]. Previous studies have shown that the temperature at the surface of the decaying needle litter of *Pinus densiflora* was a major environmental factor contributing to the changes in fungal communities [81]. Water is also indispensable for living cells. Water participates in almost all chemical reactions in vivo and plays an important role in maintaining the stability of protein macromolecules. In general, suitable humidity conditions are required for fungi growth. Mold fungi grow vigorously when the relative humidity (RH) is over 90%, while decay fungi are active at around 80% RH, whereas a few fungi, such as xerotolerant and xerophilic species, are able to grow under a relative humidity of 65%.

The temperature and availability of water are two important factors that strongly affect the fungi germination time and growth rate, but the effect of one factor cannot be entirely distinguished from another, especially in the natural environment. In this study, we used RDA analysis to reveal the relationship between microbial assemblages and each environmental factor and found that temperature contributed the most to the community. However, Zhou and Hyde [26] found that rainfall positively affected fungal occurrence on bamboo culms; however, temperature and humidity had little impact. There are two possible reasons for these apparently conflicting results. A high throughput sequencing method, instead of cultivation, was adopted in our study, which was able to observe all the microorganisms on the bamboo surface theoretically. In addition, compared with Hongkong, the temperature of our study site fluctuated more, which could have a greater impact on the fungal community. Correlation analysis showed (Figure 7b,c) that seven genera (*Sphaerulina*, *Symmetrospora*, *Monascus*, *Papiliotrema*, *Cystobasidium*, *Devonomyces*, and *Erythrobasidium*) were positively correlated with temperature, humidity, and light in the roofed groups. On the other hand, *Fusarium* was negatively correlated with these factors. Among the unroofed groups, the correlation analysis identified that four genera (*Buckleyzyma*, *Arthrinium*, *Hannaella*, and a genus of unclassified_Sordariomycetes) had a positive correlation with temperature, humidity, and light, while *Curvibasidium* showed a negative correlation with these environmental factors. Additionally, rainfall was positively correlated with *Symmetrospora* and negatively correlated with *Alternaria*, *Sphaerulina*, and a genus of unclassified_Chaetothyriales. Specifically, *Sphaerulina* was found to be positively correlated with temperature, humidity, and light, and previous studies have shown that this genus is more likely to infect plants under appropriate temperature conditions [82].

### 3.4. Function of Fungi

**Figure 8:**
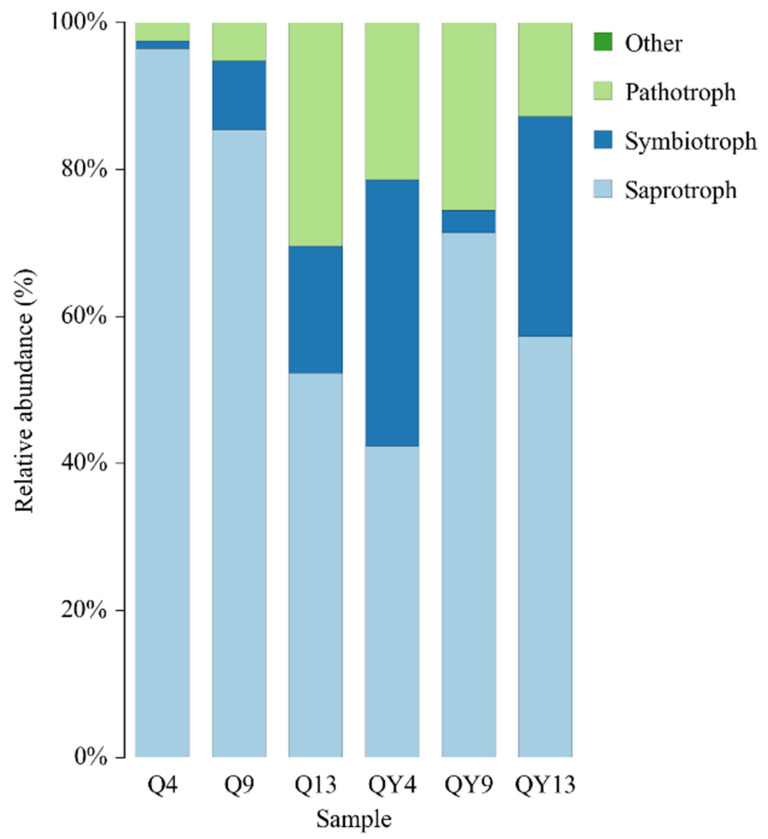
Changes in bamboo fungal functional composition in different groups at the trophic level.

The FUNGuild database was used to predict fungal functions. As shown in Figure 8 and Table S1, saprotroph was the most abundant trophic mode out of all the groups. Saprotrophs are fungi that obtain their energy by decomposing dead organic matter, such as leaf litter and wood. They play a critical role in nutrient cycling and organic matter decomposition and are important contributors to soil health. Previous studies have shown that saprotroph was the dominant trophic mode in woody debris [33], forest soil [83], and even deep-sea sediments [84]. However, saprotrophic organisms that grow on the surface of bamboo may impair its structural integrity and have a negative impact on its mechanical properties. In addition, the relative abundance of saprotrophs in unroofed groups was lower than that of roofed groups. This result is in accordance with a previous study, which reported that reduced precipitation and combined effects increased the saprotroph fungal communities [85]. Interestingly, in roofed groups, the proportion of saprotrophs showed a decreasing trend, and that of pathotroph and symbiotroph showed an increasing trend during the deterioration process. However, the relative abundance of these three trophic modes was relatively stable among the unroofed groups.

### 3.5. Chemical Composition Determination

**Figure 9:**
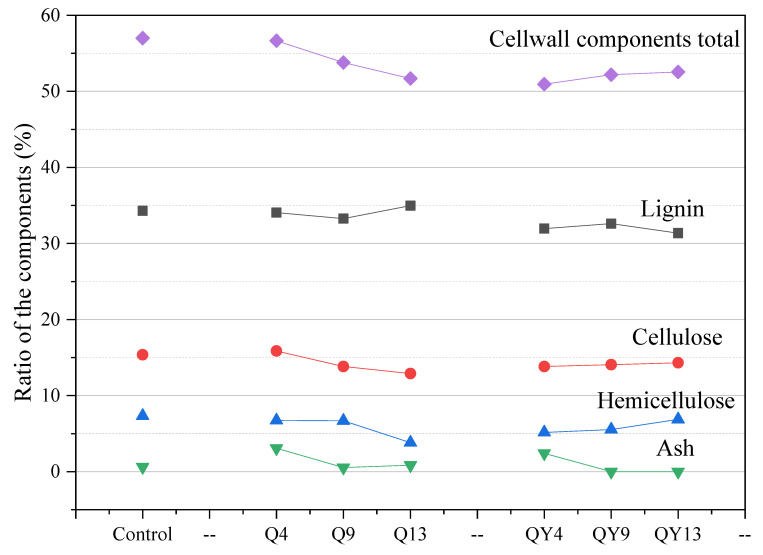
Chemical composition of bamboo green (% *wt*/*wt*, dry weight basis) during the round bamboo deterioration process.

After exposure to the roofed and unroofed environments for 4 to 13 weeks, round bamboo was attacked by various microorganisms, which inevitably affected the structure and properties of bamboo. As round bamboo for construction is generally applied in various lengths of long poles, the surface of this, commercially called bamboo green, is of great concern. Therefore, the bamboo green, 1 mm in depth, was selected to conduct the analyses of chemical composition and the determination of other related properties. The relative amount of chemical composition, including lignin, cellulose, hemicellulose, and ash, is presented in Figure 9. This research has shown that bamboo green is different from the inner culm in the percentage of the three main components, accounting for a high ratio of lignin [86]. Under both roofed and unroofed conditions, bamboo green presented a descending amount of the cell wall components total (CWT), particularly unroofed bamboo, as much as 10.6%, indicating a degradation in the bamboo cell wall from week_4 to week_13.

The CWT of the roofed bamboo samples slightly changed at week_4 and then continuously decreased, which mainly resulted from the declined ratio of cellulose and hemicellulose. As the temperature and humidity of the environment climbed over 23 °C and 80% from week_4, the community richness and activity of the fungi increased, leading to a mass loss in cell wall components. Comparatively, unroofed bamboo samples presented different changes in their chemical composition from the roofed samples. The amount of lignin, cellulose, and hemicellulose correspondingly dropped by 2.3%, 1.6%, and 2.2% during the first four weeks, leading to a significant drop in CWT. This early decomposition of the bamboo surface may have been caused by comprehensive factors, including fungal succession, light radiation, and leaching. As a kind of polyphenol, lignin is sensitive to light, especially UV light, which has been reported in many studies in the literature [87,88]. In addition, under favorable conditions, decaying fungi that exist in the environment are liable to colonize on the bamboo surface, for example, *Curvibasidium*, *Symmetrospora,* and other unidentified fungi-degrading lignin. The combined effect of light and fungi caused the degradation of lignin while leaching reduced the amount.

**Figure 10:**
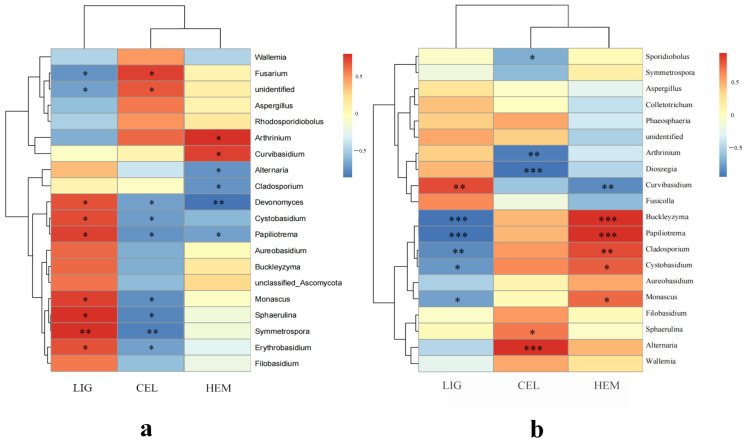
Heat map of the correlation between fungal communities and the relative content of lignin, cellulose, and hemicellulose at the genus level of the roofed (**a**) and unroofed groups (**b**) during the round bamboo deterioration process. The colors in the heat maps indicate the Spearman correlation coefficient r which ranges from −1 to 1; r < 0, negative correlation (blue); r > 0, positive correlation (red); Asterisks represent significance level: * *p* < 0.05, ** *p* < 0.01, and *** *p* < 0.001.

The correlation between the fungal community and the relative content of lignin, hemicellulose, and cellulose at the genus level was plotted to reveal the reasons for bamboo decomposition (Figure 10). In comparison with the roofed groups, the unroofed ones displayed a more significant negative correlation between the chemical components and fungal community, which explained the substantial and rapid decline in CWT. The relative content of lignin in the roofed bamboo was negatively correlated with *Fusarium* and an unidentified genus. The content of cellulose was negatively correlated with *Devonomyces*, *Cystobasidium*, *Papiliotrema*, *Monascus*, *Sphaerulina*, *Symmetrospora*, and *Erythrobasidium*. The content of hemicellulose was negatively correlated with *Alternaria*, *Cladosporium*, *Devonomyces*, and *Papiliotrema*. As for the unroofed groups, the relative content of lignin was negatively correlated with *Buckleyzyma*, *Papiliotrema*, *Cladosporium*, *Cystobasidium*, and *Monascus*. The content of cellulose was negatively correlated with *Sporidiobolus*, *Arthrinium*, and *Dioszegia*. The content of hemicellulose was negatively correlated with *Curvibasidium*. Interestingly, *Cladosporium*, as the dominant genus in each group, was negatively correlated with the content of hemicellulose in the roofed groups, whereas they presented a positive correlation with hemicellulose and a negative correlation with lignin in the unroofed groups. As mentioned above, *Cladosporium* has a broad lifestyle and is widely distributed in various bamboo materials. Furthermore, *Cladosporium* is generally associated with a necrotrophic lifestyle and has a great capability of lignocellulosic material degrading. Halaburgi et al. (2011) purified and characterized a new laccase from *Cladosporium cladosporioides* and found the potential value of this enzyme in the decolorization of polyaromatic dyes as well as in the biotransformation of phenolic compounds [89]. The lignin biodegradation of *Cladosporium* sp. could also be facilitated by a combination with carbonaceous nanomaterials [90]. Additionally, due to the abundant genes for enzymes that degrade hemicelluloses (e.g., families GH5, GH31, GH35, and GH45) in the genome of *Cladosporium* sp., it endows it with a strong capacity in hemicellulose degrading [91]. Xylan is the main component of hemicellulose. Hong et al. (2011) found that *Cladosporium cladosporioides* H1 was able to produce xylanase, and this xylanase was stable at various temperatures [92]

Therefore, we speculated that *Cladosporium* tended to produce more hemicellulose degrading enzymes, such as xylanase and mannanase, in a roofed environment but is prone to secrete more lignin-degrading enzymes (i.e., laccase, lignin peroxidase, and manganese-dependent peroxidase) in an unroofed environment; these enzymes could lead to different effects on the surface structure of bamboo. This may be the main reason for the function shift of *Cladosporium* between the roofed and unroofed environments.

### 3.6. SEM Analysis

**Figure 11:**
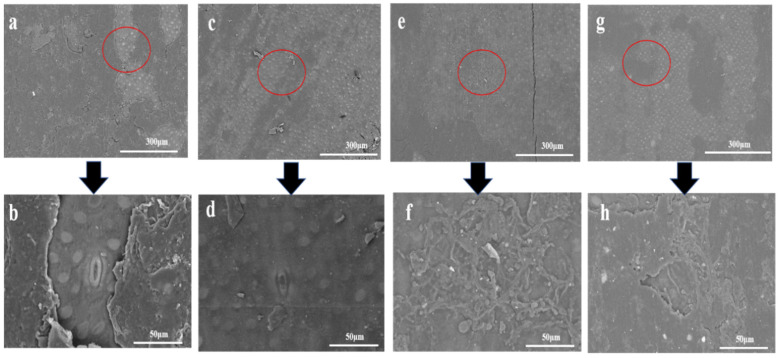
SEM images of control and roofed round bamboo samples during the deterioration process. (**a**,**b**) are the pictures of the control sample; (**c**,**d**) are the pictures of the sample from Q4 group; (**e**,**f**) are the pictures of the sample from Q9 group; (**g**,**h**) are the pictures of the sample from the Q13 group).

**Figure 12:**
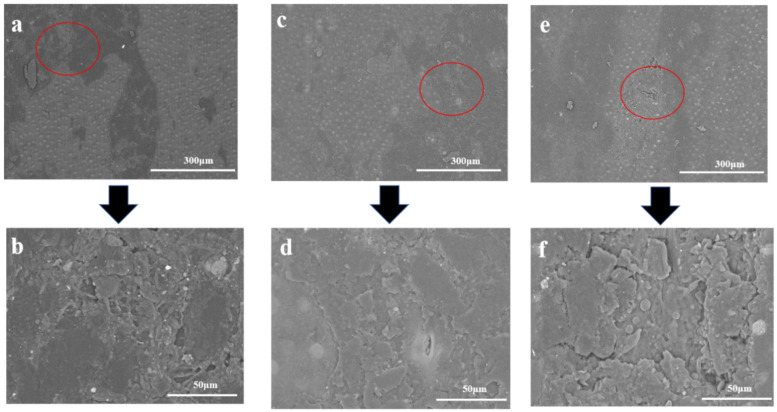
SEM images of unroofed bamboo samples during the round bamboo deterioration process. (**a**,**b**) are the pictures of sample from QY4 group; (**c**,**d**) are the pictures of sample from QY9 group; (**e**,**f**) are the pictures of sample from QY13 group).

Round bamboo, when placed outdoors, underwent environmental and microbiological effects, and the chemical components of bamboo green changed with time, which caused variation in the structure. SEM was applied to observe the morphology of bamboo green, and the results under roofed and unroofed conditions are presented in Figure 11 and Figure 12, respectively. The surface of the control samples was generally overlayed with ridges or flaky wax, and the sphere silicon cells were hidden underneath (Figure 11a,b) [93,94]. After 4 weeks of exposure, the wax on the bamboo surface, even in roofed conditions, which is free from rain and direct sunlight, became thinner or partially missing, leaving the protruding sphere silicon cells (Figure 11c,d). From week 4 to week 13, the breakage of the surface structure continued, and even the silicon cells, which were compatibly embedded in the cortical tissue, were projected with a clear interface around, which could be observed in Figure 11e–h. Moreover, hyphae began to colonize and penetrate into the bamboo surface, as shown in Figure 11f,h.

### 3.7. Contact Angle

**Figure 13:**
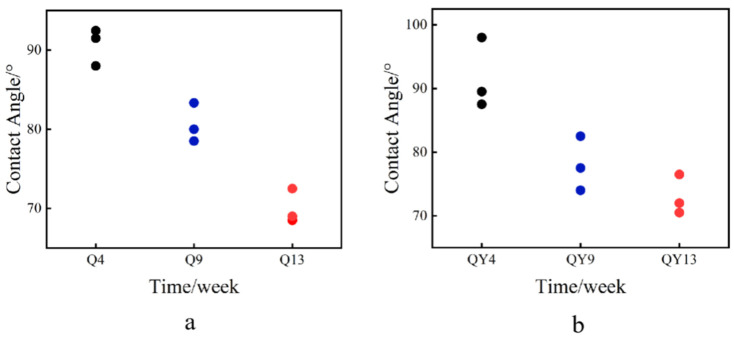
The contact angle of bamboo green during the round bamboo deterioration process. (**a**) Roofed groups; (**b**) Unroofed groups.

The contact angle of different samples was measured to investigate the variation in the hydrophobic property during deterioration. The contact angle of both the roofed and unroofed samples decreased significantly over time, indicating the declining hydrophobic property of round bamboo. As for the three main components of the bamboo cell wall, lignin was hydrophobic because of the aromatic rings [95], while cellulose and hemicellulose were hydrophilic due to the abundant hydroxyl groups. It has been reported that the contact angle of the bamboo culm decreased by 12% after degradation [96]. As shown in Figure 10, the relative content of lignin in both the roofed and unroofed groups decreased more or less during the outdoor deterioration. Therefore, we speculated that the reduction in lignin in the bamboo surface could be one of the factors causing the decline of hydrophobicity. Additionally, the partial exfoliation of the waxy layer could also contribute to the reduction in hydrophobicity, as shown in Figure 12 and Figure 13. However, the hydrophobic property of bamboo, especially in the outdoor environment, was affected by many factors, such as microbes, surface wax, and light.

## 4. Conclusions

This study demonstrated the fungal community succession and characteristics variation in round bamboo during a thirteen-week deterioration period in the typical rainy season of a subtropical monsoon climate. The diversity of bamboo fungal communities exhibited significant spatial and temporal variations, with Ascomycota and Basidiomycota being the dominant phyla in both roofed and unroofed environments throughout the deterioration period. PCoA analysis demonstrated that deterioration time had a greater impact on the succession of the fungal community on round bamboo compared to different rainfall conditions. RDA analysis further revealed that temperature was the most significant environmental factor affecting the fungal community. Chemical composition analysis indicated that lignin, cellulose, as well as hemicellulose degraded more or less in two biologically hazardous conditions during the deterioration process. Correlation analysis revealed that *Cladosporium* presented a negative correlation with hemicellulose in roofed samples but showed a positive correlation with hemicellulose and a negative correlation with lignin in the unroofed samples. The results of SEM revealed that the surface structure of round bamboo underwent continuous damage. The hydrophilic property of round bamboo increased in roofed and unroofed environments during the deterioration process. Overall, these results provided a fundamental understanding of the underlying mechanisms of the outdoor deterioration of round bamboo. However, as biodeterioration is a relatively slow process, future research should investigate the fungal community succession and characteristics variation in round bamboo on a longer timescale. Moreover, multi-omic approaches should be used to explore the specific contribution of core fungi groups on the deterioration of round bamboo.

## Data Availability

The data that support the findings of this study are available from the corresponding author upon reasonable request.

## Supplementary Materials

The following supporting information can be downloaded at: https://www.mdpi.com/article/10.3390/jof9060691/s1, Figure S1: C3.1 (a) and C3.2 (b) biologically hazardous conditions; Figure S2: Shannon index of the fungal community at different stages; Figure S3: UPGMA of the fungal community at different stages (Mantissa 1, 2, and 3 indicate repetition groups); Figure S4: XPS analysis of bamboo green C4. Table S1: Proportion of bamboo fungal functional composition in different groups at trophic level.
