# Supplementary material for: Succession of Fungal Community during Outdoor Deterioration of Round Bamboo
Source: J Fungi (Basel). 2023 Jun 20;9(6):691. doi: 10.3390/jof9060691 (PMC10301112; doi:10.3390/jof9060691)
Supplement: Supplementary file 1 [file jof-09-00691-s001.zip › jof-2427365-supplementary.pdf]

Succession of fungal community during outdoor deterioration of round bamboo

Xiaojiao An [1,†], Shuaibo Han [1,2,*,†], Xin Ren [1], John Sichone [1], Zhiwei Fan [1], Xinxing Wu [1,2], Yan Zhang [1,2], Hui Wang [1,2], Wei Cai [3] and Fangli Sun [1,2,*]

[1] School of Chemical and Materials Engineering, National Engineering & Technology Research Center for the Comprehensive Utilization of Wood-Based Resources, Zhejiang A&F University, Hangzhou 311300, China; axj624@stu.zafu.edu.cn (X.A.); rx@stu.zafu.edu.cn (X.R.); johnsichone450@gmail.com (J.S.); fzw@stu.zafu.edu.cn (Z.F.); xinxingwu@zafu.edu.cn (X.W.); zhangy@iccas.ac.cn (Y.Z.); wanghui@zafu.edu.cn (H.W.)

[2] Microbes and Insects Control Institute of Bio-based Materials, Zhejiang A&F University, Hangzhou 311300, China

[3] Anji Zhujing Bamboo Technology Co., Ltd., Huzhou 313300, China; davidcai7010052023@163.com

* Correspondence: shuaibohan@zafu.edu.cn (S.H.); 20000050@zafu.edu.cn (F.S.)

† These authors contributed equally to this work.

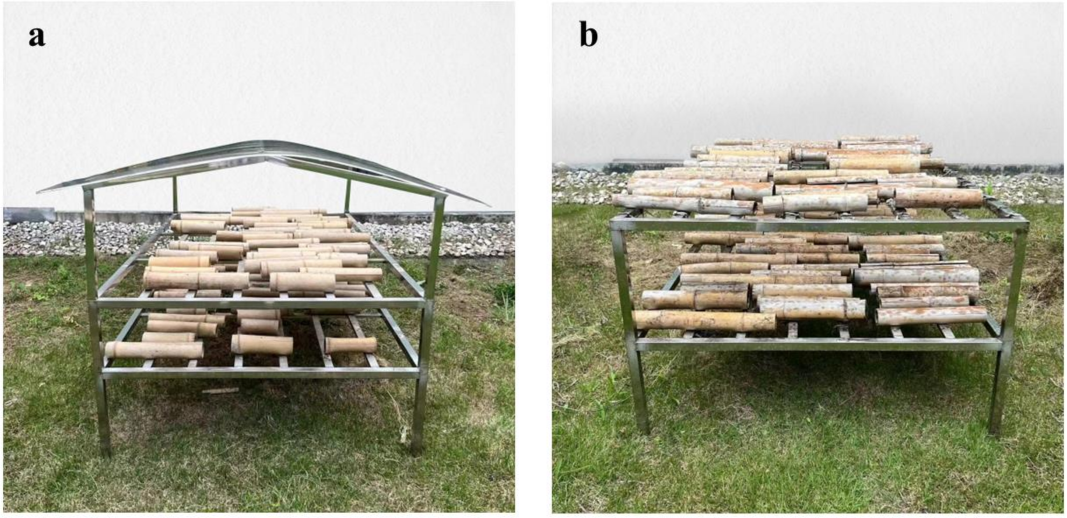

Figure S1. C3.1 (a) and C3.2 (b) biologically hazardous conditions.

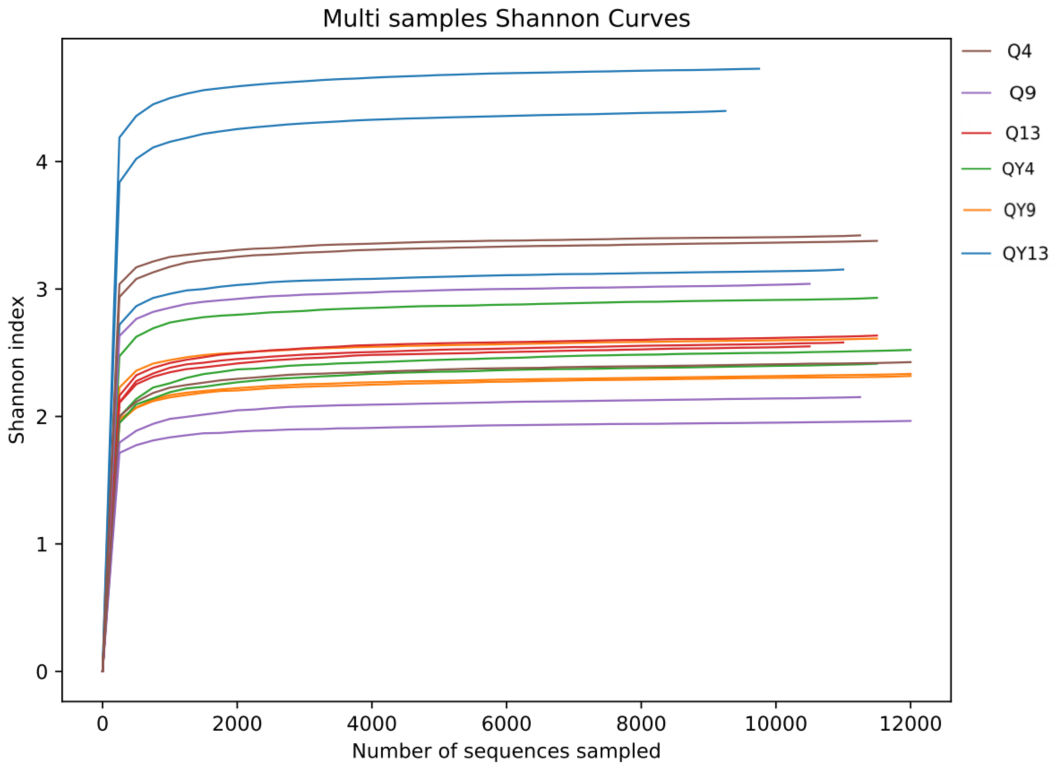

Figure S2. Shannon index of the fungal community at different stages.

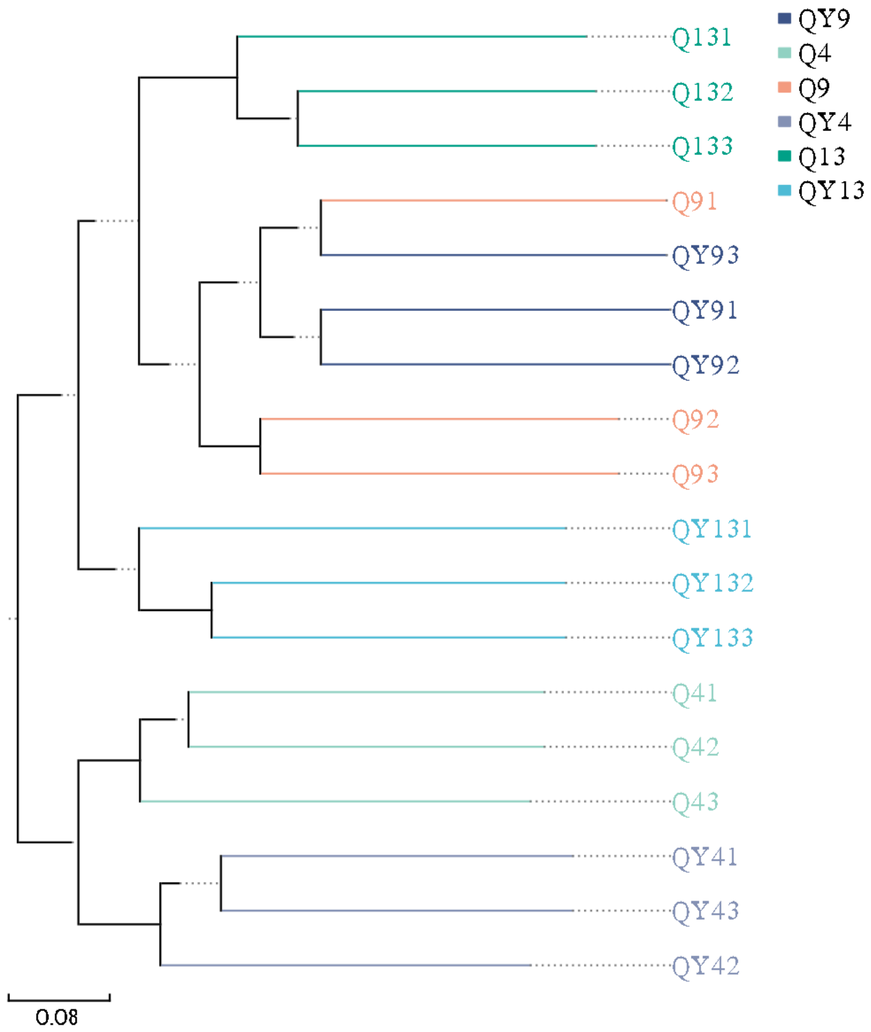

Figure S3. UPGMA of the fungal community at different stages. (Mantissa 1, 2, and 3 indicate repetition groups)

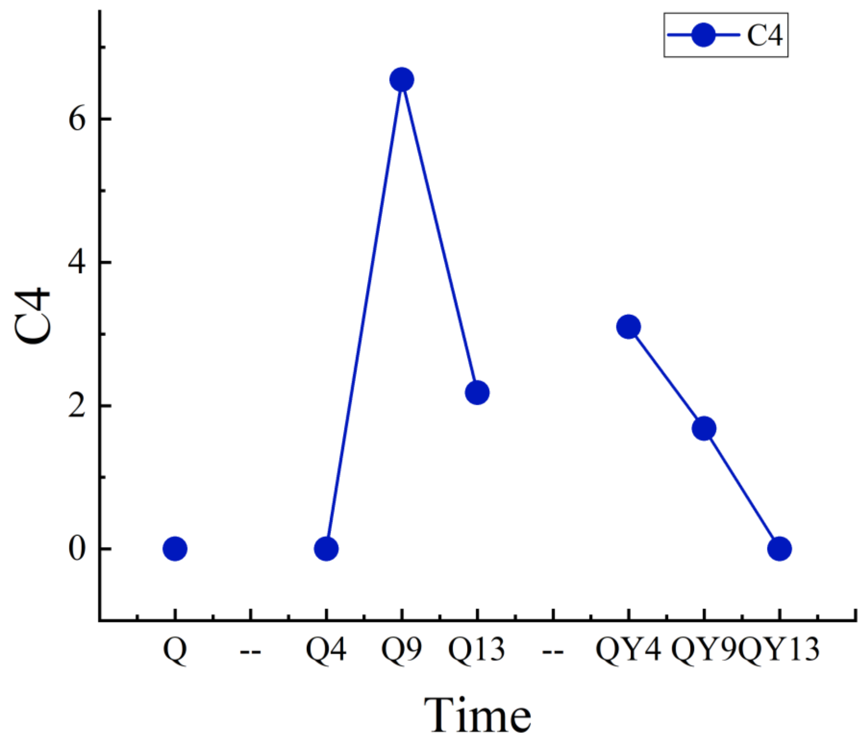

Figure S4. XPS analysis of bamboo green C4.

Table S1. Proportion of bamboo fungal functional composition in different groups at trophic level

trophic	Q4	Q9	Q13	QY4	QY9	QY13
Saprotroph	0.964787	0.854093	0.523192	0.423449	0.714228	0.573096
Symbiotroph	0.010394	0.094259	0.172593	0.362461	0.030373	0.299229
Pathotroph	0.02482	0.051649	0.304215	0.21409	0.255399	0.127675
